# Supplementary material for: Electric current control of spin helicity in an itinerant helimagnet
Source: Nat Commun. 2020 Mar 30;11:1601. doi: 10.1038/s41467-020-15380-z (PMC7105454; doi:10.1038/s41467-020-15380-z)
Supplement: Supplementary file 1 — Supplementary Information [file 41467_2020_15380_MOESM1_ESM.pdf]

Supplementary Information for
Electric current control of spin helicity in an itinerant helimagnet

Jiang et al.

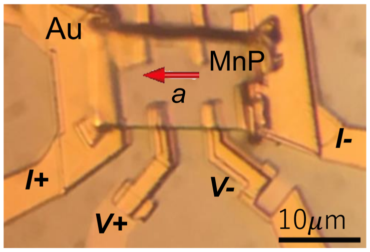

Supplementary Figure 1: An optical microscope image of the micro-fabricated MnP sample. I+, V+, V-, and I- indicate the four Au electrodes for the four-probe resistivity measurement.

Supplementary Note 1 Symmetries of helical and cycloid structures

Supplementary Figures 2 and 3 illustrate the effect of mirror operations on the helical and cycloid structures, respectively. Any mirror operation reverses the helicity of a helical structure because it has chiral symmetry. On the other hand, the cycloid structure has polar symmetry. Therefore, the spin rotation direction is unchanged by the mirrors parallel to the polarization while the perpendicular mirror reverses it.

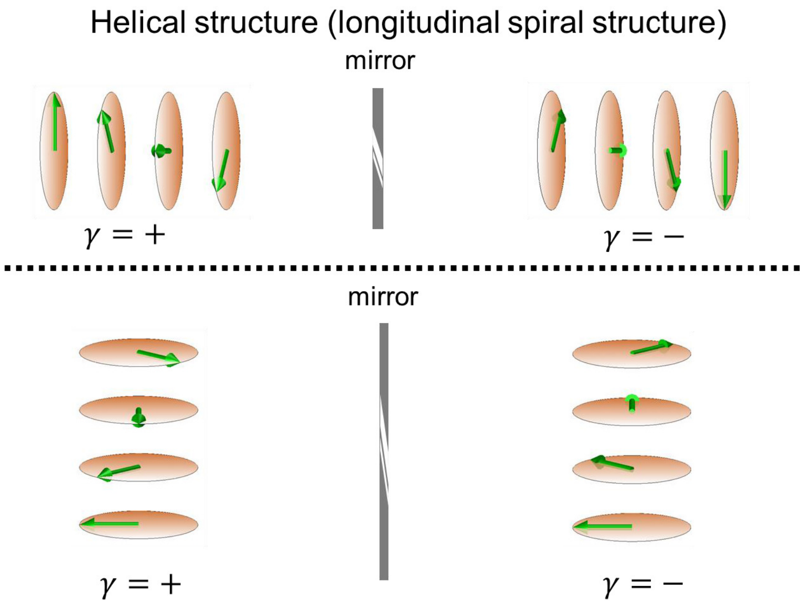

Supplementary Figure 2: The effect of mirror operations on helical structures. γ stands for the chirality.

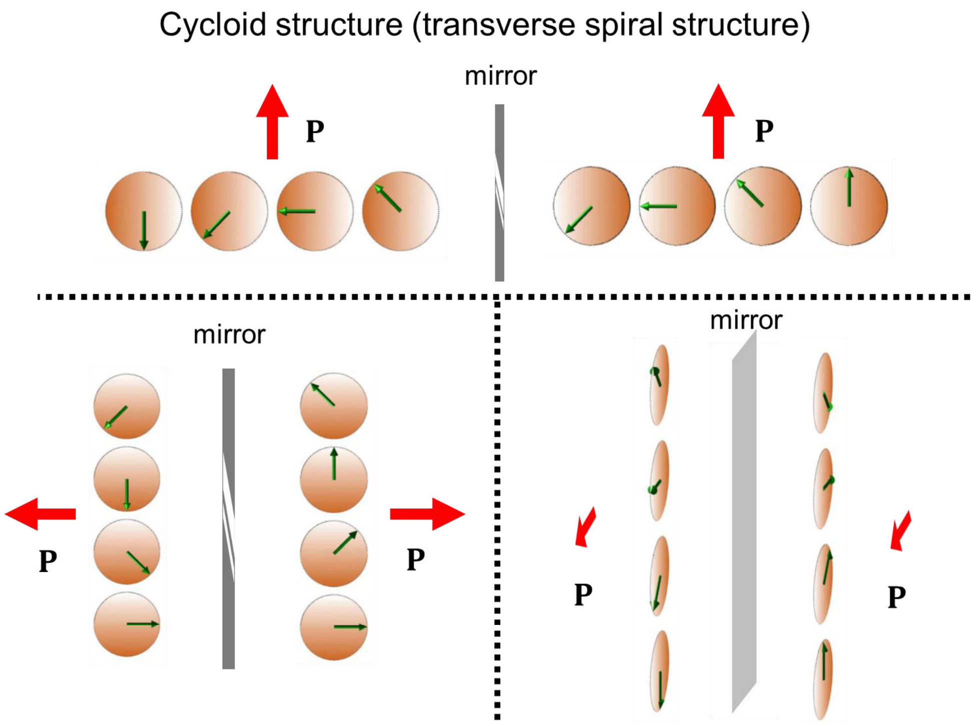

Supplementary Figure 3: The effect of mirror operations on cycloid structures. **P** stands for the electric polarization.

Supplementary Note 2   Magnetoresistance

Supplementary Figure 4 shows the magnetic field dependence of the 1st harmonic contribution of electrical resistivity at various temperatures. The magneto-structural phase transitions are observed as kinks or discontinuous changes. Large hystereses are discerned between 44 K and 60 K, reflecting the metastable nature of the magnetic state in this region. Small but finite hystereses are observed even above 60 K, in which the volume fraction of the helical magnetic phase is thought to vanish. Similar anomalous hysteresis behavior in the FM1 phase was reported in the literature[1].

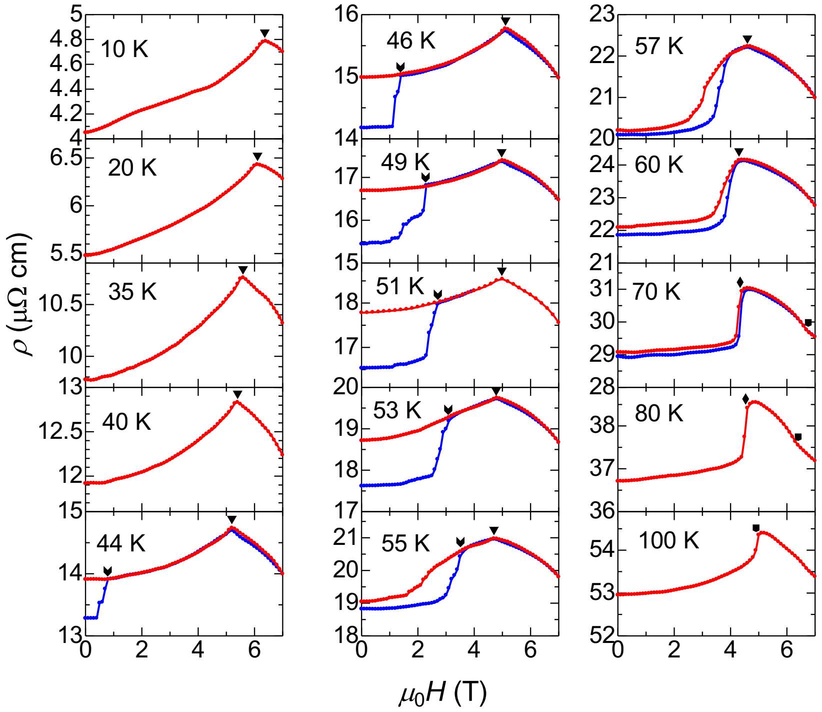

Supplementary Figure 4: Magnetic field dependence of the 1st harmonic contribution of electrical resistivity at various temperatures. The hystereses are measured between 44 K and 70 K. Before the measurement in this temperature region, the temperature is increased to 80 K and then decreased to the measured temperature in the absence of a magnetic field. The blue data shows the resistivity measured in the field-increase process just after the zero-field cool. The red curves are the data for the field-decrease process. Symbols indicate the metamagnetic transition fields shown in Fig. 2c in the main text.

Supplementary Note 3 Frequency dependence and reproducibility

At first, we measured the temperature and magnetic field dependence of ρ^{2f} at 14.3 Hz. Then, in a different experiment, we measured the frequency dependence of ρ^{2f}. The electrodes were broken and fixed between the 1st and 2nd experiments. Supplementary Figure 5 shows the magnetic field variation of $\Delta\rho^{2f} = \rho^{2f} - \rho^{2f}_{\text{offset}}$ at respective frequencies for the 1st and 2nd experiments. Here, $\rho^{2f}_{\text{offset}}$ is magnetic field independent constant and determined so that the magnetic field dependences were scaled (Supplementary Figure 5 inset). All the $\Delta\rho^{2f}$ data are almost identical to each other. This confirms the robustness of intrinsic odd-function contribution of ρ^{2f}. On the other hand, the extrinsic $\rho^{2f}_{\text{offset}}$ seems to depend on the frequency and the contact.

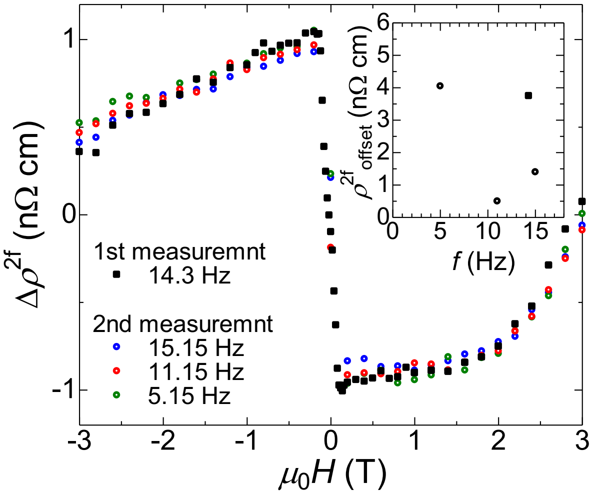

Supplementary Figure 5: Magnetic field dependence of $\Delta\rho^{2f} = \rho^{2f} - \rho^{2f}_{\text{offset}}$ at respective frequencies for the 1st and 2nd experiments (see text). The inset shows frequency dependence of $\rho^{2f}_{\text{offset}}$ for the 1st and 2nd experiments. The square dots and circles are the deta for the 1st and 2nd experiments, respectively.

Supplementary References

1. Yamazaki, T. *et al.* Novel Magnetic Chiral Structures and Unusual Temperature Hysteresis in the Metallic Helimagnet MnP. *J. Phys. Soc. Jpn.* **83**, 054711 (2014).
